# A Permutation Test for Unbalanced Paired Comparisons of Global Field Power

**DOI:** 10.1007/s10548-016-0477-3

**Published:** 2016-03-02

**Authors:** Benjamin T. Files, Vernon J. Lawhern, Anthony J. Ries, Amar R. Marathe

**Affiliations:** U.S. Army Research Laboratory, Aberdeen Proving Ground, MD USA

**Keywords:** Statistical test, Oddball paradigm, Data imbalance, Electroencephalography

## Abstract

Global field power is a valuable summary of multi-channel electroencephalography data. However, global field power is biased by the noise typical of electroencephalography experiments, so comparisons of global field power on data with unequal noise are invalid. Here, we demonstrate the relationship between the number of trials that contribute to a global field power measure and the expected value of that global field power measure. We also introduce a statistical testing procedure that can be used for multi-subject, repeated-measures (also called within-subjects) comparisons of global field power when the number of trials per condition is unequal across conditions. Simulations demonstrate the effect of unequal trial numbers on global field power comparisons and show the validity of the proposed test in contrast to conventional approaches. Finally, the proposed test and two alternative tests are applied to data collected in a rapid serial visual presentation target detection experiment. The results show that the proposed test finds global field power differences in the classical P3 range; the other tests find differences in that range but also at other times including at times before stimulus onset. These results are interpreted as showing that the proposed test is valid and sensitive to real within-subject differences in global field power in multi-subject unbalanced data.

## Introduction

Global field power (GFP) is the spatial standard deviation of a montage of average-referenced electrode voltages (Lehmann and Skrandies 1980; Skrandies 1990) and is used in analysis of electroencephalography (EEG) data. A major advantage of using GFP is that it maintains statistical power that might otherwise be lost due to the need to correct for multiple comparisons when statistically testing each electrode in a potentially large array (Hamburger and vd Burgt 1991; Koenig et al. 2011; Maris 2004; Maris and Oostenveld 2007; Skrandies 1990). GFP is spatially insensitive, so it can be used to test non-spatial hypotheses or as an initial step preceding spatial analyses that are then temporally focused (Hamburger and vd Burgt 1991; Koenig and Melie-Garcia 2010). Despite the benefits of using GFP to summarize data, computing GFP involves a non-linear transformation of the data, so caution must be taken when interpreting it (Murray et al. 2008). The purpose of this article is to call attention to a situation in which using GFP along with conventional statistical testing will lead to erroneous conclusions and to offer a statistical testing procedure that overcomes this problem.

Before addressing the main purpose of the article, a distinction must be drawn between two approaches to using GFP to summarize EEG data (Tzovara et al. 2012). The first approach is to compute the GFP of single-trial data and then average the resulting single-trial GFP measurements. The other approach is to average several trials of EEG data and compute the GFP of the resulting average. Both approaches are in use (for an example of analyses involving single-trial GFP see Wagner et al. 2014), but these two approaches estimate quantities that must be interpreted differently. The first approach computes the average GFP of a single trial. This includes the GFP of both the stimulus-locked neural activity and the electrical activity, neural or otherwise that is not time-locked to the stimulus event. The second approach computes the GFP of time-averaged data. The process of averaging will have the effect of partially cancelling out signals with a random relationship to the stimulus event and thereby enhance signals that are time-locked to the stimulus event. Confusingly, both can reasonably be called the mean GFP (or mGFP), because one is the mean of the single-trial GFPs and the other is the GFP of the mean event-related potential.

When the goal is to compare the GFP of stimulus-locked signals with as much noise (in this context, anything not time-locked to the stimulus event) eliminated as possible, a situation can arise in which the degree to which noise is eliminated is different for the two conditions under comparison (i.e. data are unbalanced). This occurs, for example, in the oddball paradigm used in traditional mismatch negativity experiments (Näätänen and Alho 1995; Näätänen et al. 2004; Stefanics et al. 2015) and P3 experiments (Picton 1992) in which a class of stimuli are presented frequently, and another class of stimuli are presented infrequently. Unbalanced data can also arise when trials are segregated based on participant responses. Because there are more trials associated with the frequent condition than the infrequent condition, more noise is eliminated from the average ERP for the frequent stimulus. When noise is just as likely to increase a measure as to decrease it, then having more or less noise in one condition or the other should not lead to systematic effects. However; GFP is a *biased* statistic, because additional noise has a tendency to increase its value. A formal demonstration that GFP is a biased statistic follows, but a more intuitive explanation is simply that since GFP is the spatial standard deviation over a set of electrodes, the larger the noise on individual channels, the greater the standard deviation will be.

We are far from the first to point out the problem of using biased statistics and unbalanced datasets. The difficulties associated with estimating component peak amplitude with unbalanced data have been previously described (Picton et al. 2000). Standard estimates of spectra and coherence are also biased (Bokil et al. 2007). Proposed solutions include discarding data to force data balance, using alternative, non-biased statistics (Picton et al. 2000; Thomas et al. 2004), or the use of procedures that correct for bias (Bokil et al. 2007). Alternative and corrected estimates are not always available, and discarding otherwise perfectly good data may sacrifice power to detect experimental effects. Here, we describe a general statistical testing procedure that uses all available data and is not susceptible to problems caused by unbalanced data. This method has been used previously (Files et al. 2013), but here we describe the method in detail with a careful examination of its validity and sensitivity. The method is closely related to permutation testing procedures that have been recommended for analysis of balanced event-related potential data (Blair and Karniski 1993; Greenblatt and Pflieger 2004; Karniski et al. 1994) and to unbalanced coherence data with one subject (Maris et al. 2007), but extensions to multi-subject paired comparisons designs have not, to our knowledge, been examined.

The testing procedure described in this paper applies to a specific combination of experimental design and comparison of interest. To pinpoint this combination of design and comparison, we refer to the experiment classification scheme of Greenblatt and Pflieger (2004; their Fig. 1, p. 227). According to that scheme, the comparison for which the unbalanced paired permutation test was designed is a two condition, paired, within-group comparison. Many tests may be used for that general comparison, but only with experimental designs that produce balanced data and/or when using summary statistics that are not biased. The unbalanced paired permutation test we describe applies to a paired, within subjects design in which trial counts in the paired conditions are imbalanced leading to a biased summary statistic. A concrete example of a design and comparison for which this test is appropriate is the typical P3/oddball design in which rare targets are embedded in a series of common distracters and the comparison of interest is the GFP evoked by targets against the GFP evoked by distracters. This design has two conditions (target, distracter), both conditions apply to every subject (the conditions are paired) and the difference in the two conditions is of interest (the comparison is within-group). This example produces unbalanced data (rare targets and frequent distracters) and is using a biased summary statistic (GFP).

**Fig. 1:**
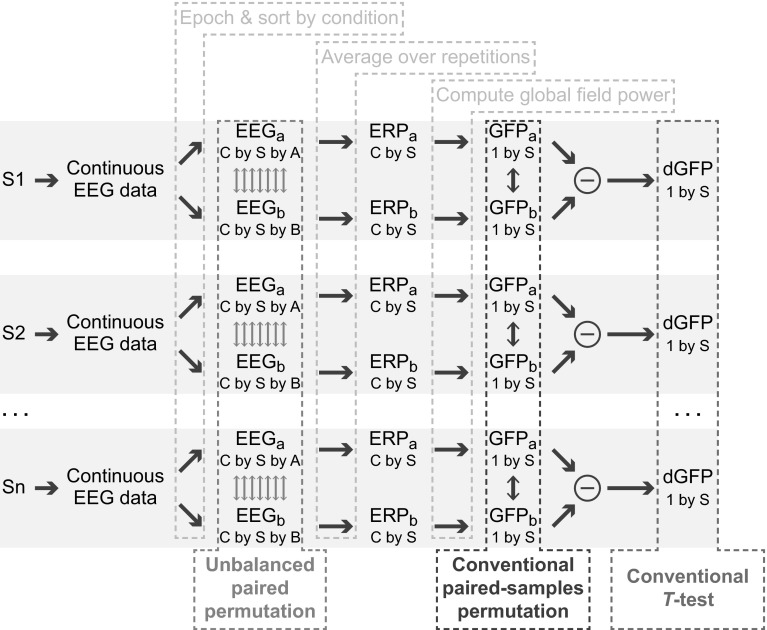
Flowchart showing the steps in computing global field power differences across two conditions. Data are obtained from subjects S1 through Sn. Continuous data are epoched around a stimulus event and sorted according to condition to obtain epoched single-trial data of dimensions *C* channels, *S* samples per epoch and A or B repetitions for conditions a and b, respectively. Average ERPs are obtained by averaging over repetitions, and then global field power is computed. The unbalanced paired permutation test carries out permutation at the single trial level, before any averaging is done. A conventional permutation test permutes after averaging and computation of global field power, and conventional *T* test would be done after computing a difference (or equivalently a paired-samples *T* test would be done on the GFPs before subtraction)

The remainder of this paper will be organized as follows. First, we show under very general assumptions that GFP is a biased statistic, and that this bias is due to non-linear effects of the number of trials used in calculating the averaged evoked potential. This finding implies that applying traditional statistical approaches for within-subjects comparisons of GFP on multi-subject data with data size imbalance will yield inaccurate results. To overcome this issue, we introduce an unbalanced paired permutation procedure that leads to a valid statistical test of GFP with unbalanced, multi-subject data. Finally, to empirically validate the proposed unbalanced paired permutation procedure, we will present simulations and analyses to illustrate the strengths and weaknesses of the unbalanced paired permutation test in comparison to alternative procedures.

## Materials and Methods

### The Expected Value of GFP

Let $Y_{ik}$, $i = 1, \ldots ,C$, $k = 1, \ldots ,K$ be from a stochastic process representing the EEG signal for channel $i$ and epoch/trial $k$ at a single time point in the epoch, where $C$ is the total number of channels and $K$ is the total number of epochs. We make the following assumptions:

$$\begin{aligned}E\left( {Y_{ik} } \right) &= \mu_{i} \\ Var\left( {Y_{ik} } \right) &= \sigma_{i}^{2} \\ Cov\left( {Y_{ik} ,Y_{im} } \right) &= 0 \\ Cov\left( {Y_{ik} ,Y_{jk} } \right) &= \rho_{ij} \end{aligned}$$

The four assumptions, taken together, mean that each channel has its own mean/variance, that non-phase-locked activity across epochs are independent and that channels within epochs are correlated as $\rho_{ij}$, with channel pairs $i$ and $j.$ We believe that assumption (4) accounts for the induced correlation due to volume conduction that is inherently present in EEG signals and that this effect is consistent across trials.

The Global Field Power (GFP) of the EEG data is given as

$$GFP = \sqrt {\mathop \sum \limits_{i = 1}^{C} \frac{{\left( {\bar{Y}_{i.} - \bar{Y}_{..} } \right)^{2} }}{C}}$$

where $\bar{Y}_{i.} = \frac{1}{K}\mathop \sum \limits_{k = 1}^{K} Y_{ik}$ is the mean of the EEG data across trials and $\bar{Y}_{..} = \frac{1}{C}\mathop \sum \limits_{i = 1}^{C} \bar{Y}_{i.}$ is the average of the EEG data over channels and over epochs. Using the Delta Method (Casella and Berger 2002), we derive an approximation (details can be found in the Appendix) to the Expected Value of the Global Field Power as:

$$E(GFP) = \sqrt {\frac{1}{C}\mathop \sum \limits_{i = 1}^{C} \left( {\frac{1}{K}\sigma_{i}^{2} + \mu_{i}^{2} } \right) - \frac{{\left( {\mathop \sum \nolimits_{i = 1}^{C} 2\mu_{i}^{2} \sigma_{i}^{2} + \frac{1}{K}\mathop \sum \nolimits_{i > j} \mu_{i} \mu_{j} \rho_{ij} } \right)}}{{K\left( {C - 1} \right)\sqrt {\left[ {\frac{1}{C - 1}\mathop \sum \nolimits_{i = 1}^{C} \left( {\frac{1}{K}\sigma_{i}^{2} + \mu_{i}^{2} } \right)} \right]^{3} } }}}$$

The sample estimates for $\sigma_{i}^{2}$, $\mu_{i}$ and $\rho_{ij}$($S^{2}$, $\bar{Y}_{i}$, and $\hat{\rho }_{ij}$) can be used in place of the population estimates above.

Crucial to our purposes here is the unavoidable factor $K$ (indicating the number of trials or epochs) which cannot be factored out of the expression, leading us to conclude that $E(GFP)$ is influenced by the number of trials used to calculate the GFP. The other factors in the expression are not problematic, either because they are expected to be consistent within a subject across typical experimental manipulations in the case of the number of channels, *C,* and the correlations between channels due to volume conductions, $\rho_{ij}$, or in the case of $\sigma_{i}^{2}$ or $\mu_{i}$ because they are potential experimental effects of interest. However, in order to draw valid inferences about potential experimental effects on GFP differences, the effect of the number of trials must be dealt with. One possibility is to ensure that the number of trials is equal across experimental conditions, but a number of experimental designs inherently produce unbalanced data. Here, we propose a statistical testing procedure that enables valid statistical testing of experimental effects in GFP with unbalanced paired data in the context of two condition, within-subjects (also called repeated measures) designs for multi-subject experiments.

### Statistical Tests for Differences in GFP

As shown in Fig. 1, in a multi-subject, two-condition, paired comparison experiment GFP is calculated for each subject/dataset by sorting epochs by condition, calculating an ERP for each condition, and then taking the root mean squared over electrodes in the condition-average ERP. To determine an average GFP difference, the subject GFP for condition A is subtracted from that of condition B, and then these differences are averaged. The question at hand is whether the GFP for condition A is statistically significantly different from the GFP for condition B.

### Conventional Tests

Conventional approaches to answering this question might employ a student’s *T* test of zero difference (equivalent to a paired-samples *T* test on the single-subject condition GFPs) or a paired-samples permutation test. Both of these conventional tests would operate on the single-subject condition GFPs. For the *T* test, the mean of the difference at each time point would be scaled by the standard error of the difference at each time point, and this value would be compared to a *T* distribution with appropriate degrees of freedom (i.e. the number of subjects minus one) to obtain a *p* value.

The paired samples permutation test is a non-parametric alternative to a paired-samples *T* test. The null hypothesis for a paired-samples permutation test is that the data labeled Condition A and the data labeled Condition B came from the same distribution. If the null hypothesis is true, then the labels on the data (A or B) are effectively arbitrary. To assess whether the observed data are compatible with this null hypothesis, a null distribution is constructed by randomly relabeling the obtained data within the experimental unit (here, a subject’s GFP for Conditions A and B). In a two-condition experiment, there are only two possible labels, and so only two possible combinations of labels: A, B or B, A. Each subjects’ data can only be labeled two ways, so there are a total of 2^*N*^ possible entries in the permutation distribution (where *N* is the number of subjects). After randomly re-labelling the data, the mean GFP difference is calculated and that difference is added to the permutation distribution. When *N* is not too large, all possible permutations can be included in the distribution, but for larger *N*s a random subset of all possible permutations are used. The actually-obtained GFP difference (i.e. with the correct labels) is then compared to the permutation distribution of GFP differences. If the summary with correct labels is extreme relative to the null distribution, then the hypothesis that the labels were essentially randomly applied to the data can be rejected.

### The Unbalanced Paired Permutation Test

The unbalanced paired permutation procedure follows the typical procedure for a permutation test, but the key development here is the selection of the experimental unit. The conventional paired-samples permutation test would use the subject’s condition GFP as the experimental unit. This poses a problem, because as we have shown in the previous section, the condition GFP depends systematically on the number of trials that comprise that condition, so when the number of trials in Condition A and B are different, the null hypothesis of the paired-samples permutation test is false due to the unequal number of trials. This tells us nothing about whether there is a difference due to the experimental manipulation itself. To get around this problem, the procedure described here reshuffles the data labels at the level of the single trial, while still respecting the relative number of trials per subject. A reference implementation is available at https://github.com/btfiles/UBpermGFP

1. For each subject
  - Count the number of trials for Condition A and B.
  - Randomly shuffle the labels (A or B) across the data for that subject, such that number of trials labeled A remains constant (as do the number of trials labeled B). Call the data with shuffled labels A′ and B′
  - Compute mean ERPs for A′ and B′
  - Compute the GFP of these ERPs, and call them GFP(A′) and GFP(B′), respectively.
  - Calculate a difference GFP, dGFP = GFP(B′) − GFP(A′).
2. Compute the group mean dGFP by averaging over all subjects.
3. Add the resulting dGFP to a null permutation distribution.

Repeat this procedure many times with a different random shuffling each time. Because there are far more possible reshufflings than could be reasonably computed, an exhaustive permutation is practically impossible. Therefore, the GFP difference computed using the true (non-shuffled) labels should also be included in the null distribution (Edgington and Onghena 2007). A two-tailed *p* value is computed in the usual way: compute the proportion of entries in the null distribution that are smaller than the entry corresponding to the actual data labels and count the proportion of entries that are larger. A two-tailed *p* value is two times the smaller of these proportions.

The novelty of this method is not in applying permutation testing to EEG data (Blair and Karniski 1993; Maris and Oostenveld 2007). The unbalanced paired permutation test is a modification of standard techniques that lets it be used in tests of mGFP with unbalanced data. The goal is to show that this method allows valid statistical testing of unbalanced mGFP data, in contrast to conventional methods.

## Simulation and Experimental Methods

Three experiments were run to examine how unbalanced data can be summarized and tested using GFP. The first experiment was a simulation designed to test the relative performance of the paired-samples *T* test, the conventional paired-samples permutation test, and the unbalanced paired permutation test in a situation in which the null hypothesis was true, because labeling of the data was random. The second experiment was a simulation with the goal of assessing the sensitivity of the unbalanced paired permutation test by adding a known effect to data that were in other aspects not different. The third experiment used a GFP analysis on unbalanced data that had been previously shown to have an experimental effect using conventional ERP analysis methods.

All three experiments used data from an EEG dataset that has been described in previous publications (Cecotti et al. 2015; Marathe et al. 2015; Ries et al. 2013). Briefly, these data were collected in a rapid serial visual presentation target detection paradigm from 16 subjects (13 male, mean age 33.5 years). Target stimuli were people in simulated natural scenes holding guns, and the non-target stimuli were simulated natural scenes with no people. Participants were asked to silently count the number of targets. Electrophysiological recordings were sampled at 256 Hz from 64 active scalp electrodes and analyzed using an average reference. Large artifacts from EMG were removed through visual inspection and EOG artifacts were removed using independent component analysis (ICA), (Jung et al. 2000) prior to analysis. The voluntary, fully-informed written consent of participants in this research was obtained as required by federal and U.S. Army regulations (U. S. Department of Defense Office of the Secretary of Defense 1999; U. S. Department of the Army 1990). The investigator adhered to Army policies for the protection of human subjects (U. S. Department of the Army 1990). All human subjects testing was approved by the Institutional Review Board of the United States Army Research Laboratory.

For Experiments 1 and 2, EEG data were filtered using a high-pass filter at 1 Hz and only non-target epochs were used. Epochs extended from 1 s before to 2 s after non-target stimulus onset. To avoid contamination of non-target epochs with activity due to temporally adjacent target stimuli, only non-target epochs that were at least 3 s removed from any target stimuli were used. Epochs with any voltage exceeding ±75 μV were rejected; this left 3 of the 16 subjects with less than 150 accepted non-target epochs. These participants’ data were not used. In the 13 remaining data sets, the average number of accepted background epochs was 177.7 (std 11.2, min 153, max 189).

Experiment 3 used both target and non-target epochs. Processing of the data for Experiment 3 differed slightly from processing for Experiments 1 and 2. Specifically, epochs were required to be 2 (rather than 3) seconds away from any target stimuli for inclusion, and data were high-pass filtered at .2 Hz. The narrower exclusion window was chosen to increase the number of non-target epochs and the lower high-pass filter was chosen to avoid filtering out the low frequency P3 effect while still eliminating drift over the course of an epoch. All other processing steps were identical to those used for Experiments 1 and 2. After epoching and rejection, the average number of background epochs was 425.5 (std 83.6, min 240 max 496) and the average number of target epochs was 48.5 (std 9.4, min 25, max 54).

### Experiment 1

This experiment was a simulation intended to determine the validity of four different statistical tests on GFP summaries of unbalanced data. Validity is the criterion that when the null hypothesis is true, the probability of getting a *p* value less than or equal to alpha is equal to alpha. In other words, the probability of a false rejection should be equal to the threshold for rejection. The data used in this simulation was chosen to be typical of an EEG experiment with no experimental effects (i.e. the null hypothesis of no difference between conditions is true). To achieve this, a subset of non-target epochs were randomly selected from each subject and labeled as Condition A. The remaining non-target epochs were labeled as Condition B. These labeled trials were then submitted to statistical testing by the paired-samples *T* test (two-tailed, *df* = 12), the conventional paired-samples permutation test (with 2000 resamplings), and the unbalanced paired permutation test (with 2000 resamplings). This procedure (i.e. randomly selecting a subset and then statistical testing) was repeated 100 times in order to gather summary statistics on the false positive rate of each method. Additionally, the size of the subset of trials labeled Condition A relative to the size of the total number of trials was systematically varied, such that 1/15, 1/10, 1/8, 1/5 or 1/2 of the trials were labeled as Condition A.

To statistically test for validity, the false positive rate with a threshold for significance of .05 was compared to the nominal value (i.e. .05) for each testing method and each ratio. Two-tailed tests were used because false positive rates that are significantly higher than the nominal value indicate an overly liberal test and false positive rates that are significantly lower than the nominal value indicate an overly conservative test.

### Experiment 2

This simulation tested the sensitivity of the unbalanced paired permutation test to experimental effects. The approach was to randomly select 30 of the background epochs as Condition A and then select from the remaining background trials as Condition B. In this experiment, as in Experiment 1, the number of trials selected for Condition B was systematically varied, such that 1/2, 1/3, 1/4, and 1/5 of the trials in the experiment were labeled as Condition A. A simulated experimental effect was added to the trials in Condition A. The simulated experimental effect was a simple additive offset that was positive for half of the electrodes and negative for the other half. This simulated effect was chosen not to be similar to actual ERP effects, but instead to be simple to describe and control. The size of the effect was varied to produce a range of simulated signal-to-noise ratios. The effect powers used were (in RMS microvolts) 1.17, 1.47, 1.76, 2.05, 2.35, 2.64, 2.93, 3.25, and 3.59.

Signal-to-noise ratio was computed as the root mean squared effect divided by the root mean squared noise. Noise was defined as the residual of the non-target epochs once the mean of the non-target epochs was subtracted. This simulation was repeated 100 times in order to collect summary statistics on the true positive rate of the unbalanced paired permutation test. For each simulation, a sigmoid was fit to the sensitivity curve and was summarized as the (interpolated) SNR at which sensitivity was .5.

### Experiment 3

The goal of this experiment was to apply the three methods described above to an experimental dataset. The GFP from the target and non-target epochs was compared. Previous studies have shown differences between rare target and frequent non-target epochs using conventional ERP methods (e.g. Hamburger and vd Burgt 1991).

All three methods were applied to the experimental data with the same settings as in the simulation in Experiment 1. Results are presented with timepoint-by-timepoint test results both before and after correction for multiple comparisons. Several options are available for correcting time-series data for multiple comparisons (Groppe et al. 2011a). The approach we used was to control the false discovery rate (Benjamini and Hochberg 1995; Benjamini and Yekutieli 2001), because it is convenient to apply to all of the statistical tests under comparison without being too conservative.

As an additional comparison, we performed EEG channel-based testing using the mass univariate toolbox (Groppe et al. 2011a, b) to perform a permutation *T* test for differences in the mean ERP for targets vs. non-targets. Because both the mass univariate and GFP tests are permutation tests, we used two permutation-based methods for correcting for multiple comparisons for each test. One was a cluster-based correction (Bullmore et al. 1999), which has high power for detecting broadly distributed effects, but does not provide strong family-wise error rate control. For the ERP test, a cluster *t*-mass correction was applied with an initial inclusion criterion of .05 and an electrode adjacency criterion of 5.24 cm. For the GFP test, a cluster size (i.e. number of consecutive tests with uncorrected *p* value <.05) correction was applied with an inclusion criterion of .05. Cluster size was used instead of cluster mass, because the null distribution of cluster mass was not symmetrical about zero. The other correction provided strong control over family-wise error rate. For the ERP test, a t_max_ correction (Blair and Karniski 1993) was used. For the GFP test a similar procedure was used, but GFP difference (rather than a *t* statistic) was computed.

In summary, Experiment 1 was a simulation examining the validity of the unbalanced paired permutation test as compared with conventional paired-samples permutation test and the conventional paired *T* test under varying levels of data imbalance with a true null hypothesis. Experiment 2 was a simulation examining the sensitivity of the unbalanced paired permutation test with a false null hypothesis under varying effect sizes and data imbalances. Experiment 3 uses the three statistical tests to analyze an experimental (rather than simulated) data set.

## Results

### Experiment 1

**Fig. 2:**
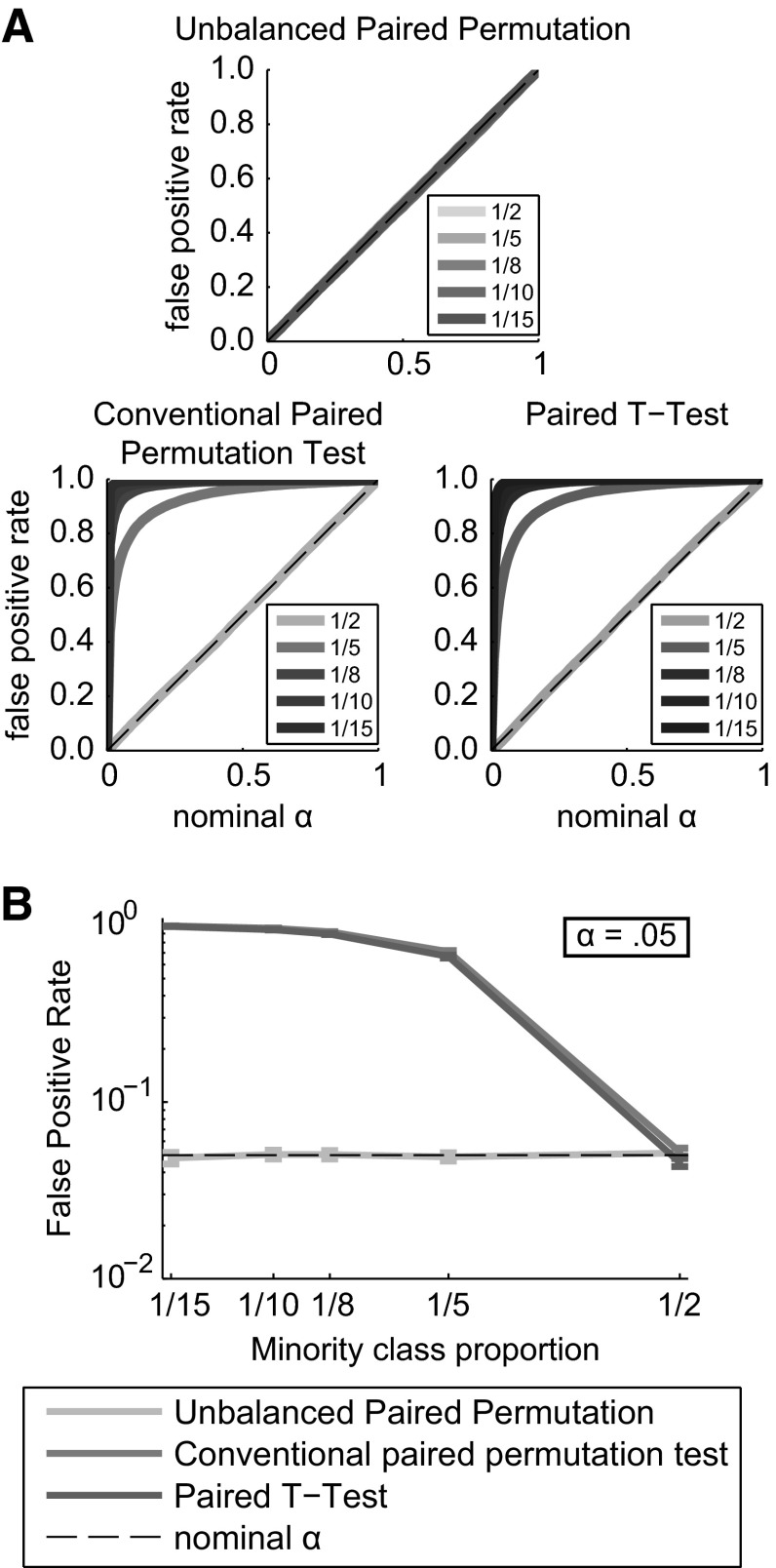
False positive statistical results under a true null hypothesis. **a** *Each panel* shows a family of validity curves that show the mean false positive rate versus significance threshold taken over 250 simulations. Data imbalance increases as curves go from *light to dark*, with the lightest curve showing balanced data through the darkest in which 1/15th of the trials are assigned to Condition A. For the unbalanced paired permutation test, all *curves* fall on the unity line. **b** Shows the comparison of false positive rates for the three statistical tests across all imbalances tested when the threshold for significance is .05. Note the log scale. The *dashed line* shows a false positive rate of .05. *Error bars* show plus and minus 1.96 standard errors of the mean

This simulation tested the validity of three statistical methods for detecting GFP difference when they were applied to unbalanced datasets. Datasets were constructed such that the null hypothesis of no difference between data labeled Condition A and data labeled Condition B was true, but the relative number of trials in Condition A and Condition B were unbalanced. A balanced dataset was also included. All methods except the unbalanced paired permutation method had false positive rates substantially above the criterion for significance (i.e. alpha) over the entire range of criteria for all but the balanced data (Fig. 2a). At alpha = .05 (Fig. 2b), all methods except the unbalanced paired permutation method had false alarm rates above the nominal value of .05 when data were unbalanced, as assessed by one-sample *T* tests (*df* = 99), all *p* < 10^−4^. When data were balanced, however, the proportion of false positives was less than .05 for the paired-samples *T* test, *T*(99) = −3.7, M = .046, 95 % CI (.042, .049), *p* = .0003.

For the unbalanced paired permutation test, mean false positive rates (with 95 % confidence intervals, Bonferroni corrected) were .052 (.046, .056), .049 (.045, .053), .050 (.046, .055), .051 (.046, .055), and .048 (.043, .054) when 1/2, 1/5, 1/8 1/10 and 1/15 of the trials were assigned to Condition A, respectively. These results show that when data are simulated under a true null hypothesis and with imbalanced data, conventional statistical tests produce more false positives than expected given any particular criterion for statistical significance. The unbalanced paired permutation test can be called valid because it produces a false positive rate equal to the chosen threshold for statistical significance.

### Experiment 2

This simulation tested the sensitivity of the unbalanced paired permutation GFP test by partitioning data for each subject into two unequal sets and then adding an artificial effect to the smaller set (labeled Condition A). The size of the added effect was varied to test the validity of the unbalanced paired permutation GFP test across a range of effect sizes. No effect was added to the other set, labeled Condition B. The number of trials in Set B was varied in order to examine sensitivity with different levels of imbalance.

**Fig. 3:**
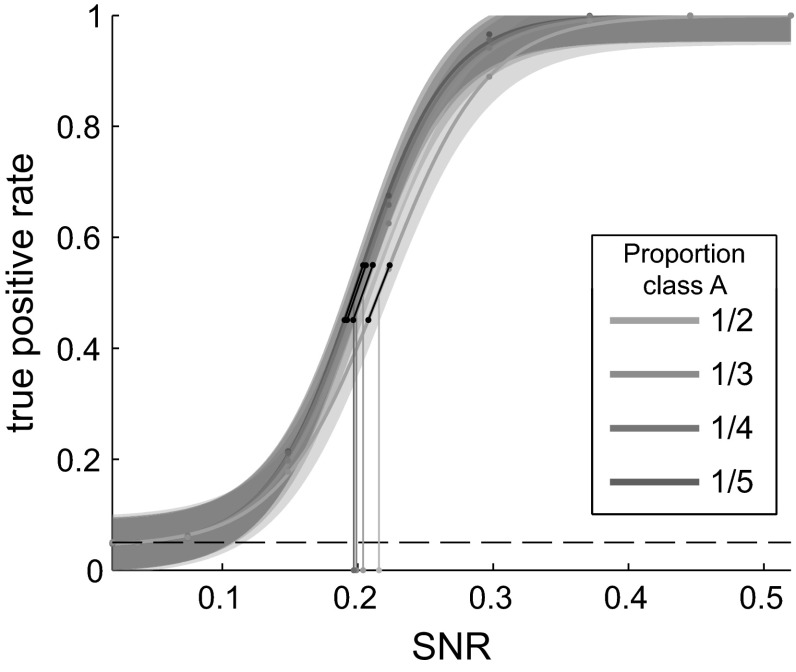
Sensitivity for the unbalanced paired permutation test with fixed number of target trials. Points show the mean proportion of tests rejecting the null hypothesis (i.e. the true positive rate) for each effect size tested for each of the four imbalances tested. *Curves* are fitted logistic functions. Highlighted areas around the curve show the 95 % confidence area for new observations. *Vertical lines* call out the SNR at which half of tests rejected the null hypothesis and *black* segments show the slope of the curve at that point. For all proportions, the number of class A trials was fixed at 30 and the number of class B trials was adjusted to achieved the proportion being tested

Across all imbalances tested, the sensitivity, as measured by true positive rate, increased with increasing effect size (Fig. 3). To assess whether changing the number of trials in Condition B while holding the number of trials in Condition A constant had an effect on the sensitivity of the unbalanced paired permutation test, a generalized linear model with a modified logit link function was fit to the sensitivity data with effect SNR and data imbalance ratio as predictors. The model included an intercept term and terms for SNR, imbalance ratio, and the interaction of SNR with imbalance ratio. Through backward elimination it was determined that the interaction term was not significant (*p* > .05), so we reduced the model to one including only SNR and imbalance ratio. The model (*df* = 3597) fit the data better than an intercept-only model (χ^2^ = 3.4 × 10^3^, *p* < 10^−10^). The effect of SNR (B = 30.7, SE = 1.54, *T* = 19.9, *p* = 2.9 × 10^−88^) was statistically significant. The effect of ratio (i.e. the proportion of trials that were targets) was statistically significant (B = −2.2, SE = .73, *T* = −3.04, *p* = .0024). So, although the biggest factor for the sensitivity of the test is the SNR of the effect in question, increasing the number of trials in the no-effect condition and thereby decreasing the proportion of trials that are in the effect condition increases sensitivity as well.

In summary, Experiment 2 shows that the unbalanced paired permutation test increases its sensitivity as the effect size in the affected condition increases, but increasing the number of trials in the majority, non-affected condition also has a relatively small but positive impact on the sensitivity of the test.

### Experiment 3

**Fig. 4:**
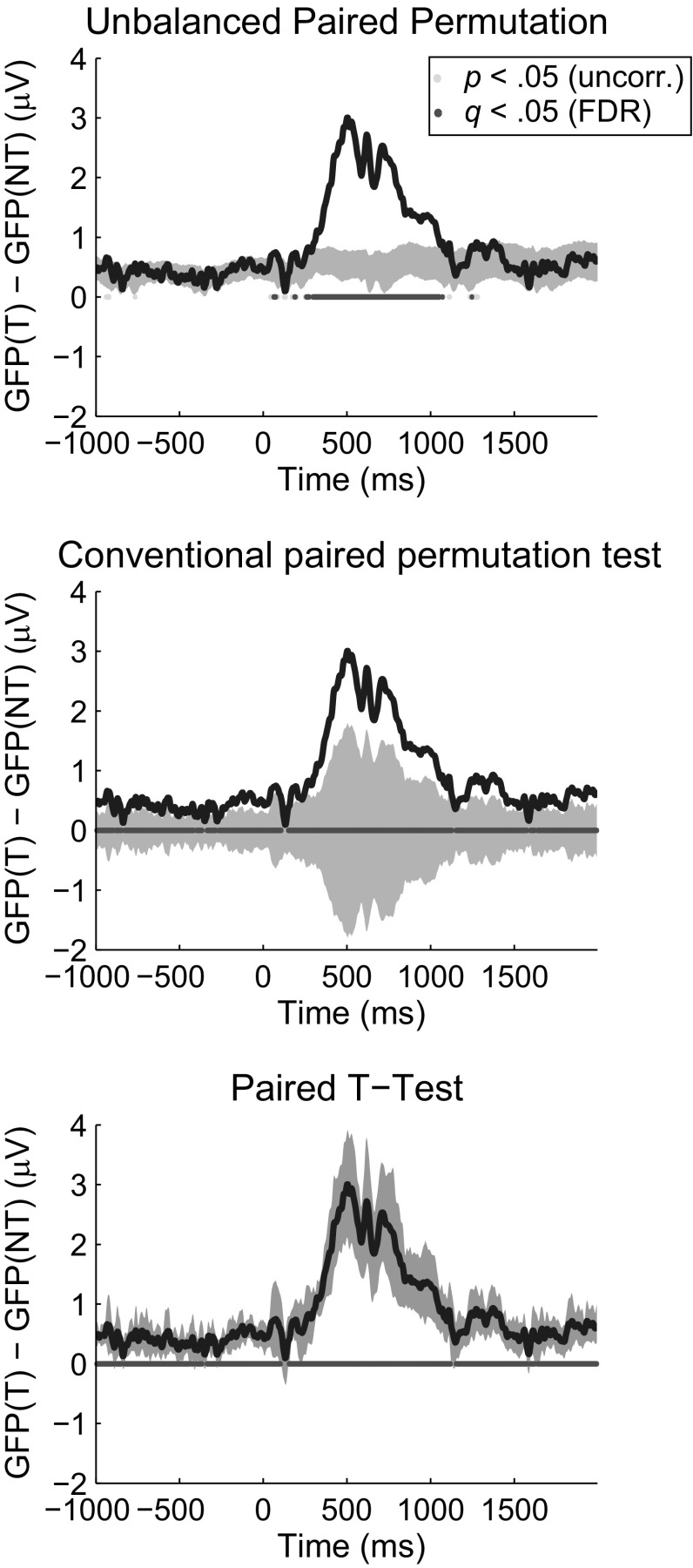
Results of three statistical tests applied to experimental data. In all panels, the *dark blue line* depicts the measured group mean GFP difference of Condition A, target images, minus Condition B, background images. The three tests are the unbalanced paired permutation test, conventional paired *T* test and the conventional paired permutation test. For the two permutation tests, the shaded area covers the central 95 % of the permutation distribution. Values falling outside of that area correspond to a rejection of the null hypothesis at the uncorrected .05 criterion. For the *T* test, the shaded area depicts a 95 % confidence interval about the measured value. Samples at which the confidence area does not include 0 correspond to a rejection of the null hypothesis at the uncorrected .05 criterion. For all tests, *pale* marks at y = 0 indicate samples at which the null hypothesis is rejected at the uncorrected .05 criterion, and *dark* marks indicate samples at which the null hypothesis is rejected after FDR correction. All tests were done with N = 13 subjects

This experiment used EEG data from 13 subjects performing a target detection task in a rapid serial visual presentation paradigm. After artifact rejection, an average of 48.5 (range 25–54) target trials and 426.1 (range 240–496) non-target trials were maintained. In the terms used in Experiments 1 and 2, data imbalance as the proportion of *target* trials was 1/9.8 on average (range 1/8.8–1/10.6). The three statistical tests (unbalanced paired permutation, conventional paired-samples permutation, paired-samples *T* test) were applied to the GFP at each time point from 1000 ms before stimulus onset to 2000 ms after onset. At the sampling rate of 256 Hz, this results in 768 time points. Results of the four statistical tests are shown in Fig. 4. All three tests rejected their respective null hypotheses (*q* < .05, FDR corrected) from 296 to 1051 ms post-stimulus onset. However, consistent with the simulation results from Experiment 1, the conventional statistical tests rejected their respective null hypotheses prior to stimulus onset 239 and 235 times out of 256 total pre-stimulus samples for paired-samples *T* test and paired-samples permutation test, respectively.

Because these analyses were carried out on experimental (rather than simulated) data, it is impossible to differentiate false positive results from true positive results. With that said, the most likely explanation for rejections of the null hypothesis preceding stimulus onset is that these rejections are false positives. This is because a stimulus preceding a target and a stimulus preceding a non-target were always background stimuli, so it is more likely that rejections of the null hypothesis preceding the target onset are false positives caused by data imbalance. The same argument cannot be made for rejections after stimulus onset by the conventional methods. Those rejections could be false positives due to the sensitivity of the tests to data imbalance demonstrated in Experiments 1 and 2, or they could be reflecting a true, meaningful difference in the GFP caused by persistent differences in neural processing of the target stimulus. In that case, the failure of the unbalanced paired permutation test to reject would be considered a false negative. Generally, nonparametric methods such as permutation tests are less sensitive than their parametric counterparts if the assumptions of the parametric test are met. However, the simulation results illustrating the sensitivity of the conventional methods to data imbalance show that at best the post-stimulus rejections of the conventional statistical tests are equivocal.

**Fig. 5:**
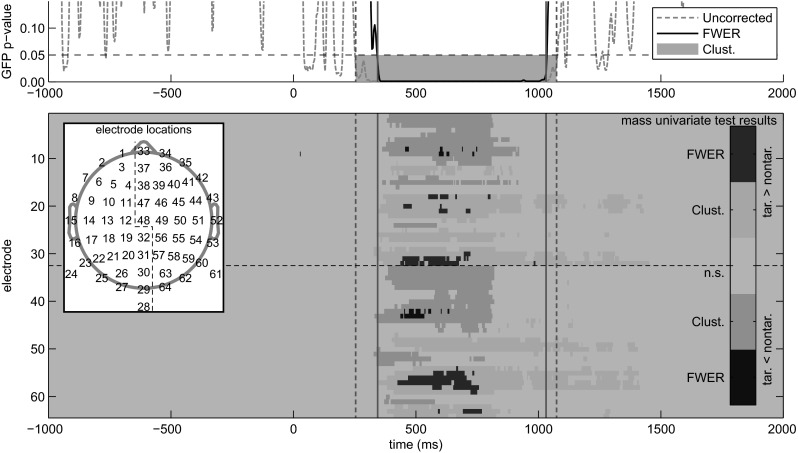
Global field power and mass univariate analyses. The upper marginal plot shows *p* values from the unbalanced paired permutation test. The uncorrected *p* values are obtained directly from the test, and strong family-wise error rate (FWER) control was achieved using a modified maximum statistic. The *pink* area indicates a cluster of consecutive results that was identified as statistically significant using cluster-size correction. The main panel shows statistical results for mass univariate mean ERP analyses with one row per EEG electrode. The inlayed diagram shows a mapping of electrode number to approximate position on the head. *Dark red* and *dark blue* indicate significant positive and negative differences (target minus non-target) as determined using *t*_max_ correction to achieve strong FWER control (*p* < .05). *Pale pink* and *blue* indicate clusters of positive and negative differences, respectively, with *p* < .05 determined using cluster mass correction. *Vertical green bars* indicate the time windows over which the GFP testing found a statistically reliable difference using strong FWER and cluster-based statistics for *solid* and *dashed bars*, respectively. All tests were done with N = 13 subjects

For comparison with the within-subject GFP difference testing, we also carried out a mass univariate test that would be used to simultaneously test hypotheses about both spatial and temporal effects. These tests clearly are testing for different kinds of effects, so a direct comparison of their results is not possible, but looking at them simultaneously reveals what can be gained from GFP analysis relative to mass univariate analysis of mean ERP (Fig. 5). The GFP test, under both strong and weak family-wise error correction for multiple comparisons, finds statistically reliable differences in the time-range of the P3. The mass univariate ERP test also finds effects in that time range. Under the cluster-based correction, both positive and negative clusters are found that start well after the GFP cluster but also extend past the GFP cluster. Under strong FWER control, focal effects are found at the peaks of the differences identified by the cluster method. As might be expected due to performing one test per time point (rather than one test per channel per time point), GFP testing appears to be more sensitive after correction for multiple comparisons than mass univariate testing, but some caveats must be kept in mind. First, because these are experimental data, we lack ground truth and cannot determine with certainty which rejections of the null hypothesis are correct. Second, using cluster-based corrections for multiple comparisons provides *p* values for clusters as a whole, so particular claims about parts of clusters, such as their onsets and offsets, are made with caution. Finally, GFP and ERP tests are looking for different effects, but they are not mutually exclusive. If both temporal and spatial hypotheses are of interest, a temporal window could be established by GFP testing, and then mass univariate ERP tests could be performed over the time window(s) identified in GFP testing.

## Discussion

Global field power (Lehmann and Skrandies 1980; Skrandies 1990) offers a useful summary of multichannel EEG data (Koenig et al. 2011; Murray et al. 2008). Because the value of GFP depends on the variability of the data used to construct the GFP, GFP comparisons are problematic in common EEG protocols that result in unbalanced datasets. The unbalanced paired permutation test described here aims to solve that problem. Experiment 1 showed that, in contrast to other methods, the unbalanced paired permutation test is valid when applied to unbalanced datasets. In particular, the false positive rates of the paired-samples *T* test and the paired-samples permutation test are higher than the nominal alpha criterion when data are imbalanced. Experiment 2 simulated EEG effects of various sizes and showed that the unbalanced paired permutation test is able to detect simulated experimental effects over a range of effect sizes. This result would be expected for any valid statistical test. Perhaps more interesting is that increasing the number of trials in the majority class (i.e. by increasing the data imbalance) improves the sensitivity of the unbalanced paired permutation test, although this effect was small relative to the effect of increasing the size of the effect. So, in contrast to conventional tests that were impacted negatively by data imbalance, the unbalanced paired permutation test proposed here takes advantage of the additional samples in an unbalanced data set. Experiment 3 applied all three statistical tests to an experimental (rather than simulated) data set. Because ground truth is unknown, rejections of the null hypothesis cannot be classified with certainty as false or true rejections. The conventional statistical tests rejected the null hypothesis of equal GFP during the pre-stimulus interval, and the most likely explanation is that these rejections were false rejections. Overall, the unbalanced paired permutation test performs well when applied to global field power calculated on within-subjects, unbalanced data.

Unbalanced data also present concerns when interpreting conventional (e.g. ANOVA) analyses of ERP amplitude, which is why the mean amplitude over a time window is recommended rather than the absolute maximum amplitude (Luck 2005; Picton et al. 2000). This differs from the current approach to GFP in that mean amplitude is not biased with increased noise levels. However, there is not always an a priori reason to choose one time window over another when analyzing ERP amplitude. In these situations, the unbalanced paired permutation test described here can be used to establish time windows of interest for subsequent ERP analyses.

Computing GFP difference is one of many approaches to reducing comparisons over an entire EEG array to a single statistic. Other approaches include global dissimilarity (Lehmann and Skrandies 1980) and taking the GFP of an ERP difference (Greenblatt and Pflieger 2004). The resampling strategy employed by the unbalanced paired permutation test described here could be adapted to these (and any other) summary statistics. If the summary statistic is known to be unbiased it may not be worth the additional computational effort to apply this resampling scheme, but we expect the unbalanced paired permutation test should be valid for unbiased statistics as well.

The problems with using biased summaries of unbalanced data have been noted in the context of coherence, a common measure of the association of two time-varying signals (Bokil et al. 2007; Maris et al. 2007). Maris and colleagues applied a permutation test to unbalanced, single-subject data. A similar approach has been applied to single-subject GFP (Wagner et al. 2014). The key difference between those permutation tests and the one described here lies in extending the method to multi-subject designs. The unbalanced paired permutation test introduced here can be applied directly to multi-subject designs and yields a useful summary of the entirety of the data, without having to consider individual subjects in isolation or having to discard useful data. As such, the unbalanced paired permutation test should be a useful tool when applied to data gathered under paradigms that are unbalanced by design, as those that evoke P3 (Picton 1992) or mismatch negativity (Näätänen and Alho 1995; Stefanics et al. 2015) components as well as data sorted by behavioral responses.

## Appendix: Derivation of the Expected Value of the Global Field Power

The global field power (GFP) of the EEG data is given as

$$GFP = \sqrt {\mathop \sum \limits_{i = 1}^{C} \frac{{\left( {\bar{Y}_{i.} - \bar{Y}_{..} } \right)^{2} }}{C}}$$

where $\bar{Y}_{i.} = \frac{1}{K}\mathop \sum \limits_{k = 1}^{K} Y_{ik}$ is the mean of the EEG data across trials and $\bar{Y}_{..} = \frac{1}{C}\mathop \sum \limits_{i = 1}^{C} \bar{Y}_{..}$ is the average of the EEG data over channels and over epochs. For ease of notation, define $X_{i} = \bar{Y}_{i.}$ and $\overline{X} = \bar{Y}_{..}$. If we assume the EEG is globally average referenced, the GFP reduces to:

$$GFP = \sqrt {\mathop \sum \limits_{i = 1}^{C} \frac{{\left( {X_{i} } \right)^{2} }}{C}}$$

Note that the GFP can be re-written in terms of the sample variance $S^{2}$ as

$$\frac{C}{C - 1}GFP^{2} = \frac{C}{C - 1}\left[ {\mathop \sum \limits_{i = 1}^{C} \frac{{\left( {X_{i} } \right)^{2} }}{C}} \right] = S^{2}$$

which can be further rewritten as

$$GFP = \sqrt {\frac{C - 1}{C}S^{2} }$$

We are interested in $E\left( {GFP} \right)$, the expected value of the GFP. From the expressions above, we need to calculate $E\left( {\sqrt {S^{2} } } \right)$, the expectation of the square root of the sample variance. Without any distributional assumptions on the distribution of $S^{2}$ this expression has no closed form solution. However, this expression is of the form $E(f\left( x \right))$, where $f\left( x \right) = \sqrt x$. Therefore, we can use a Taylor Series approximation to derive an estimate for the mean and variance of $E\left( {\sqrt {S^{2} } } \right)$.

For background material, the Taylor Series of a function $f\left( \cdot \right)$ around a value $a$ is:

$$f\left( x \right) = f\left( a \right) + f^{'} \left( a \right)\left( {x - a} \right) + f^{''} \left( a \right)\frac{{\left( {x - a} \right)^{2} }}{2!} + \ldots$$

The second-order Taylor Series approximation is the first three terms of this sum:

$$f\left( x \right) \approx f\left( a \right) + f^{'} \left( a \right)\left( {x - a} \right) + f^{''} \left( a \right)\frac{{\left( {x - a} \right)^{2} }}{2}$$

If we set $a = E\left( {S^{2} } \right)$, $f(x) = \sqrt x$, and let the random variable $x = S^{2} ,$ then $E(f\left( x \right))$ simplifies to

$$E\left( {\sqrt {S^{2} } } \right) \approx f\left( a \right) + f^{''} \left( a \right)\frac{{E\left( {\left( {S^{2} - E(S^{2} )} \right)^{2} } \right)}}{2} = \sqrt {E(S^{2} )} - \frac{1}{{8\sqrt {E\left( {S^{2} } \right)^{3} } }}{\text{Var}}\left( {{\text{S}}^{2} } \right)$$

Now note that $S^{2} = \frac{1}{C - 1}\mathop \sum \limits_{i = 1}^{C} \left( {X_{i} } \right)^{2}$. First, we need

$$\begin{aligned}{E\left( {S^{2} } \right)} &  = \frac{1}{C - 1}E\left( {\mathop \sum \limits_{i = 1}^{C} X_{i}^{2} } \right) \\ &  = \frac{1}{C - 1}\mathop \sum \limits_{i = 1}^{C} E\left( {X_{i}^{2} } \right) \\  &  = \frac{1}{C - 1}\mathop \sum \limits_{i = 1}^{C} \left( {Var\left( {X_{i} } \right) + \left[ {E\left( {X_{i} } \right)} \right]^{2} } \right) \end{aligned}$$

For $$Var\left( {S^{2} } \right)$$:

$$Var\left( {S^{2} } \right) = \frac{1}{{\left( {C - 1} \right)^{2} }}Var\left( {\mathop \sum \limits_{i = 1}^{C} X_{i}^{2} } \right) = \frac{1}{{\left( {C - 1} \right)^{2} }}\mathop \sum \limits_{i = 1}^{C} Var\left( {X_{i}^{2} } \right) + 2\frac{1}{{\left( {C - 1} \right)^{2} }}\mathop \sum \limits_{i > j} Cov(X_{i}^{2} ,X_{j}^{2} )$$

For the variance, we can use a first-order Taylor Series approximation (referred to as the *Delta Method* (Casella and Berger 2002) in the statistics literature), with $f\left( x \right) = x^{2} ,\;a = E\left( {X_{i} } \right) = \frac{1}{K}\mathop \sum \limits_{k = 1}^{K} E(Y_{ik} ) = \mu_{i}$:

$$ \begin{aligned}&{\frac{1}{{(C - 1)^{2} }}\mathop \sum \limits_{i = 1}^{C} Var\left( {X_{i}^{2} } \right)}  + 2\frac{1}{{(C - 1)^{2} }}\mathop \sum \limits_{i > j} Cov\left( {X_{i}^{2} ,X_{j}^{2} } \right) \\  & \quad \approx \frac{4}{{(C - 1)^{2} }}\left[ {\mathop \sum \limits_{i = 1}^{C} \mu_{i}^{2} Var\left( {X_{i} } \right)} \right] + \frac{8}{{(C - 1)^{2} }}\mathop \sum \limits_{i > j} \mu_{i} \mu_{j} Cov\left( {X_{i} ,X_{j} } \right) \end{aligned} $$

Now note that $X_{i} = \frac{1}{K}\mathop \sum \limits_{k = 1}^{K} Y_{ik}$, the average of the EEG data per channel over all epochs. Using this, the expectation becomes:

$$\begin{aligned}{E(S^{2})} & = \frac{1}{C - 1}\mathop \sum \limits_{i = 1}^{C} \left( {Var\left( {X_{i} } \right) + \left[ {E\left( {X_{i} } \right)} \right]^{2}} \right) \\ {E(S^{2} )} &  = \frac{1}{C - 1}\mathop \sum \limits_{i = 1}^{C} \frac{1}{{K^{2} }}Var\left( {\mathop \sum \limits_{k = 1}^{K} Y_{ik} } \right) + \frac{1}{{K^{2} }}\left[ {E\left( {\mathop \sum \limits_{k = 1}^{K} Y_{ik} } \right)} \right]^{2}  \\ {\;E(S^{2} )} &  = \frac{1}{C - 1}\mathop \sum \limits_{i = 1}^{C} \frac{1}{K}\sigma_{i}^{2} + \mu_{i}^{2}  \end{aligned}$$

Similarly,

$$\begin{aligned} {Var\left( {S^{2} } \right)} &  = \frac{4}{{\left( {C - 1} \right)^{2} }}\left[ {\mathop \sum \limits_{i = 1}^{C} \mu_{i}^{2} Var\left( {X_{i} } \right)} \right] + \frac{8}{{(C - 1)^{2} }}\mathop \sum \limits_{i > j} \mu_{i} \mu_{j} Cov\left( {X_{i} ,X_{j} } \right) \\ &  = \frac{4}{{\left( {C - 1} \right)^{2} }}\left[ {\mathop \sum \limits_{i = 1}^{C} \mu_{i}^{2} Var\left( {\frac{1}{K}\mathop \sum \limits_{k = 1}^{K} Y_{ik} } \right)} \right] + \frac{8}{{(C - 1)^{2} }}\mathop \sum \limits_{i > j} \mu_{i} \mu_{j} Cov\left( {\frac{1}{K}\mathop \sum \limits_{k = 1}^{K} Y_{ik} ,\frac{1}{K}\mathop \sum \limits_{k = 1}^{K} Y_{jk} } \right) \\ &  = \frac{4}{{K\left( {C - 1} \right)^{2} }}\left[ {\mathop \sum \limits_{i = 1}^{C} \mu_{i}^{2} \sigma_{i}^{2} } \right] + \frac{8}{{K^{2} (C - 1)^{2} }}\mathop \sum \limits_{i > j} \mu_{i} \mu_{j} \rho_{ij} \end{aligned}$$

In summary,

$$E\left( {GFP} \right) = \sqrt {\frac{C - 1}{C}} * {\text{E}}\left( {\sqrt {S^{2} } } \right) = \sqrt {\frac{1}{C}\mathop \sum \limits_{i = 1}^{C} \left( {\frac{1}{K}\sigma_{i}^{2} + \mu_{i}^{2} } \right) - \frac{{\left( {\mathop \sum \nolimits_{i = 1}^{C} 2\mu_{i}^{2} \sigma_{i}^{2} + \frac{1}{K}\mathop \sum \nolimits_{i > j} \mu_{i} \mu_{j} \rho_{ij} } \right)}}{{K\left( {C - 1} \right)\sqrt {\left[ {\frac{1}{C - 1}\mathop \sum \nolimits_{i = 1}^{C} \left( {\frac{1}{K}\sigma_{i}^{2} + \mu_{i}^{2} } \right)} \right]^{3} } }}}$$
